# A rapid and reference-free imputation method for low-cost genotyping platforms

**DOI:** 10.1038/s41598-023-50086-4

**Published:** 2023-12-27

**Authors:** Vinh Chi Duong, Giang Minh Vu, Thien Khac Nguyen, Hung Tran The Nguyen, Thang Luong Pham, Nam S. Vo, Tham Hong Hoang

**Affiliations:** 1 Center for Biomedical Informatics, Vingroup Big Data Institute, Hanoi, Vietnam; 2 GeneStory Joint Stock Company, Hanoi, Vietnam; 3 Nanyang Technological University, Singapore, Singapore

**Keywords:** Computational models, Machine learning, Microarrays

## Abstract

Most current genotype imputation methods are reference-based, which posed several challenges to users, such as high computational costs and reference panel inaccessibility. Thus, deep learning models are expected to create reference-free imputation methods performing with higher accuracy and shortening the running time. We proposed a imputation method using recurrent neural networks integrating with an additional discriminator network, namely GRUD. This method was applied to datasets from genotyping chips and Low-Pass Whole Genome Sequencing (LP-WGS) with the reference panels from The 1000 Genomes Project (1KGP) phase 3, the dataset of 4810 Singaporeans (SG10K), and The 1000 Vietnamese Genome Project (VN1K). Our model performed more accurately than other existing methods on multiple datasets, especially with common variants with large minor allele frequency, and shrank running time and memory usage. In summary, these results indicated that GRUD can be implemented in genomic analyses to improve the accuracy and running-time of genotype imputation.

## Introduction

In addition to whole-genome sequencing (WGS), due to reasonable cost and scalability, SNP microarray-based technology and low-pass sequencing has been widely used for obtaining genotype information in genome-wide association studies (GWAS)^1^, estimation of polygenic risk scores (PRS)^2^, and trait heritability analysis^3^. However, to identify more loci, it is required larger sample size, more sequencing depth for low-pass sequencing, and a denser SNP array for microarray-based genotyping. Recently, this conundrum can be resolved by genotyping imputation, which predicts untyped variants using a haplotype reference panel. Most existing genotype imputation methods are reference-based, and the idea behind these methods is that people from the same or similar ancestor might share small sections of DNA sequence between them, thus, by considering phased genotypes obtained using SNP array or low-pass sequencing and the recombinations of haplotypes presenting in the haplotype reference panel, it is possible to predict the genotypes of unobserved variants. Hence, such approaches require a large-scale reference panel^4^. Over the years, several methods have been developed by using this approach with the main algorithm being the Hidden Markov model (HMM) (i.e. IMPUTE5^5^, BEAGLE5^6^, Minimac4^7^, MACH^8^, and fastPHASE^9^). However, these methods posed a number of challenges to users, such as high computational costs for applying a large number of samples for genotype calling and problems related to reference panel inaccessibility for public research use.

Machine learning and deep learning models, which have been significantly improved in various fields, offered an alternative solution applied in genotype imputation in order to create reference-free methods performing with higher accuracy and shortening the running time^10–12^. Kojima et al.^13^ proposed a bi-directional recurrent neural network (RNN)-based imputation technique that uses phased genotype as input data to estimate allele probabilities for unobserved variants. Despite the fact that the imputation accuracy of Kojima’s model was shown to be equivalent to other conventional methods, its running-time has been significantly higher compared to others. This model’s imputation process takes approximately two times as long as using IMPUTE2, and has been around 50 times higher than Minimac3^13^.

In this paper, we proposed a deep learning-based imputation method called GRUD with the generator using GRU for RNN cells, couple with a sequential layer into the generator model to extract features for input vector patterns of bidirectional RNN. Also, we added a discriminator network based on generative adversarial network (GAN)^14^ model to enhance running-time, and imputation accuracy. Haplotype datasets from the 1000 Genomes Project (1KGP) phase 3^15^, SG10K^16^, and 1000 Vietnamese Genomes Project (VN1K) (https://genome.vinbigdata.org), along with inhouse and simulated datasets from SNP microarray chips and low-pass sequencing were used to assess the performance of GRUD in the comparison with other existing techniques.

## Results

### Evaluation with 1KGP datasets

#### Infinium Omni2.5-8 Beadchip

**Figure 1:**
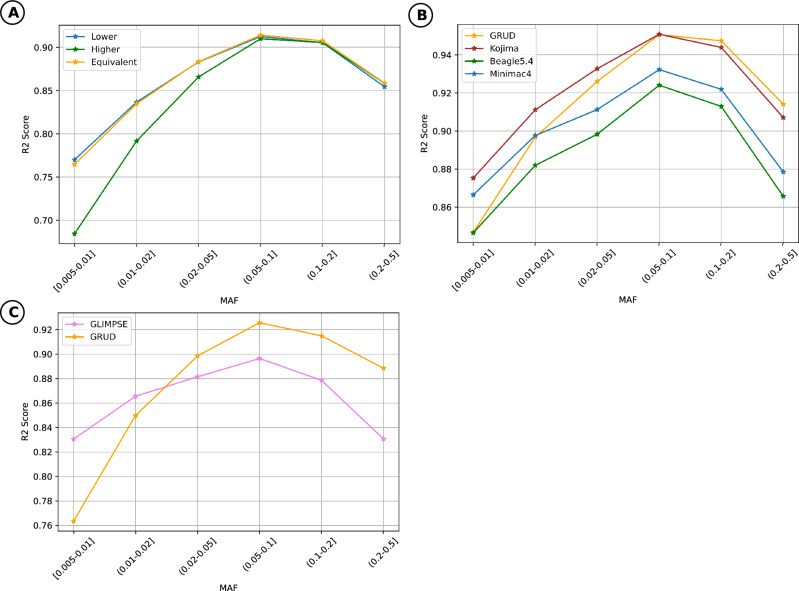
Accuracy assessment of GRUD model with 1000 genomes project phase 3 (1KGP3) data compared to other existing methods. (**A**) Comparison of $R^2$ in MAF scales between different settings of the model namely Higher, Lower and Equivalent on 1KGP3 chromosome 22 (Omni2.5). Higher: the model trained with higher priority to high MAF; Lower: the model trained with higher priority to low MAF; Equivalent: the model trained with no priority to MAF. (**B**) Comparison of $R^2$ values in linear MAF scale between the proposed method, Minimac4, Kojima, and Beagle5.4 imputation methods for 100 individuals in 1KGP3 on chromosome 22 (**C**) Comparison of $R^2$ values between the proposed method and GLIMPSE for 21 individuals on chromosome 22 of 1KGP3 dataset. MAF: Minor allele frequency; $R^2$: Squared Pearson correlation.

Among different setting of loss functions of the generator, squared Pearson’s correlation between allele dosages and true genotype counts-$R^2$ calculated on MAF bins greater than 0.005 of the Equivalent model showed a similar pattern with the Lower model, which were significantly higher than that from the Higher model. Hence, the Equivalent model will be chosen to be evaluated in comparison with the other existing methods (Fig. 1A) (GRUD model, in short).

We used Minimac4, Beagle5.4 as the existing imputation techniques based on Li and Stephen model, and a deep-learning model of Kojima, to compare the imputation performance of our proposed model. The comparison of $R^2$ values obtained by the squared correlation of WGS data and allele dosages from imputed genotype was displayed in Fig. 1B. The imputation accuracy for variants with MAF > 0.05 was slightly higher than Kojima, and significantly higher than Beagle5.4 and Minimac4. In terms of variants with MAF$\le$0.05, the $R^2$ of our proposed model was higher or comparable to those of Beagle5.4 and Minimac4, but it was lower than Kojima. Overall, GRUD method (average $R^2$=0.819, overall $R^2$=0.948) performed comparably with Kojima (average $R^2$=0.831, overall $R^2$=0.946) (Table 1).

Regarding average memory usage, GRUD model only consumed 0.4389 GiB, nearly seven-time less than that of Kojima (2.8215 GiB) (Table 2) on Intel$\circledR$ Core™ i9-9900K CPU @ 3.60GHz $\times$ 16 and 62,7 GiB memory in a thread. In addition, the running-time of GRUD model was 743s, which was significantly shorter than Kojima’s (35,082s). It also should be noted that the running time of both methods depend on libraries and computational processors. In general, GRUD model was faster and took less resource than the Kojima method with default settings.

#### Low-pass whole genome sequencing

We employed GLIMPSE as a representative of imputation and phasing methods designed for low-coverage sequencing data, and compared the imputation accuracy of our model and this method (Fig. 1C). Generally, the performance of GRUD on low-pass data was better than GLIMPSE, particularly for the variants with MAF>0.02. The average $R^2$ per variant of GRUD and GLIMPSE was 0.0818234 and 0.165236, respectively (Table 1).

### Evaluation with SG10K datasets

**Figure 2:**
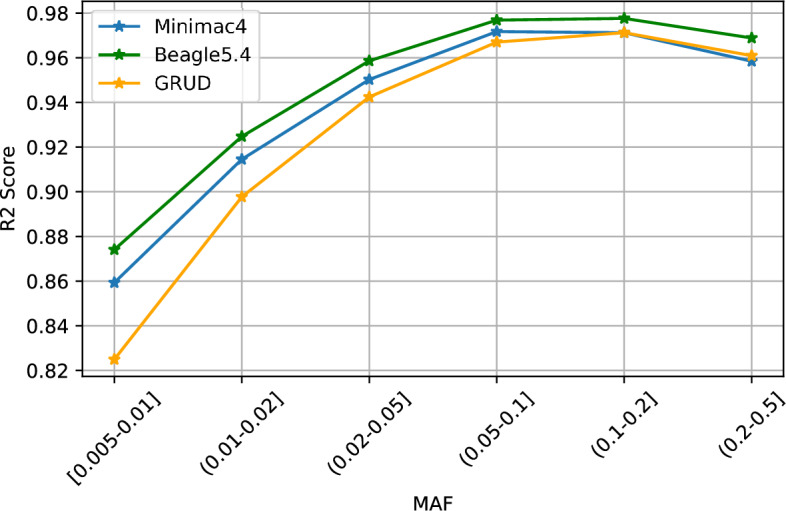
Accuracy assessment of GRUD model with SG10K data compared to other existing methods. *MAF* Minor allele frequency, $R^2$ Squared Pearson correlation.

The comparison of performance between GRUD and HMM-based models on SG10K dataset was depicted in Fig. 2. The imputation accuracy of GRUD (average $R^2$=0.116065, overall $R^2$=0.966) was comparable with those of Beagle5.4 (average $R^2$=0.23131, overall $R^2$=0.974) and Minimac4 (average $R^2$=0.22661, overall $R^2$=0.967) (Table 1). Concerning variants with MAF$\le$0.05, the $R^2$ value of GRUD was slightly lower compared to those of Beagle5.4 and Minimac4.

### Evaluation with Vietnamese datasets

**Figure 3:**
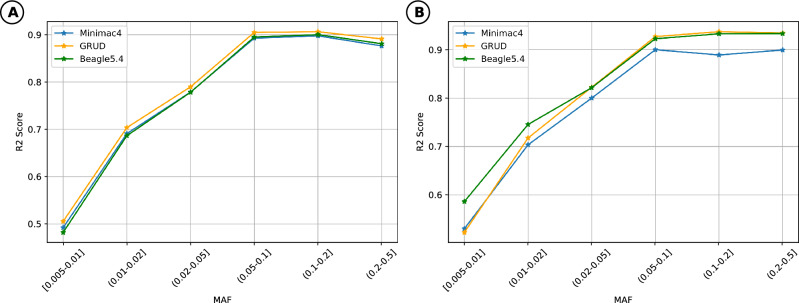
Accuracy assessment of GRUD model with VN1K data compared to other existing methods. (**A**) The comparison of $R^2$ values between our method, Minimac4 and Beagle5.4 on chromosome 22 of the GSAv3 dataset (24 samples). (**B**) The accuracy of $R^2$ values between our method, Minimac4 and Beagle5.4 on chromosome 22 of the APMRA dataset (94 samples). *MAF* Minor allele frequency, $R^2$ Squared Pearson correlation.

Regarding other SNP array platforms, we assessed the imputation performance of GRUD model on 2 Vietnamese genotype datasets generated by GSAv3 (Fig. 3A) and APMRA (Fig. 3B) chip, using VN1K as a reference panel. In general, the $R^2$ values of GRUD(average $R^2$=0.416114, overall $R^2$=0.94462) was not inferior to those of Beagle5.4(average $R^2$=0.240624, overall $R^2$=0.94349) and Minimac4(average $R^2$=0.238438, overall $R^2$=0.91792). Specifically, in the experiments with 94 samples genotyped by APMRA, the $R^2$ values of GRUD were similar to those of Minimac4 and were slightly lower than Beagle5.4 for variants with MAF$\le$ 0.02, conversely with variants with MAF>0.05, the $R^2$ of GRUD are approximate to Beagle5.4 and higher than Minimac4. Meanwhile, for 24 samples genotyped by GSAv3, the imputation accuracy of GRUD(average $R^2$=0.145081, overall $R^2$=0.918) was comparable with the Beagle5.4 (average $R^2$=0.16551, overall $R^2$=0.943) and Minimac4 (average $R^2$=0.168622, overall $R^2$=0.909) (Table 1).

## Discussion

By applying deep learning in the field of genotype imputation, we developed a new method namely GRUD to tackle the existing issues of other available methods. Our pre-trained model could be applied directly to new samples to impute genotypes, while the other HMM models have to compute parameters for each scratch once a new batch is given. We exploited the sequential layer whose ability to learn non-linear combinations and to transform input data into gaussian distribution, which could facilitate the learning process of the model, enhancing the ability to mimic real values. Furthermore, the additional discriminator network serves as a supportive model during the training process, this provides feedback to the generator to improve the accuracy and training time by detecting mistakes in the generated data to fix in the next iteration. Due to the exchange information between two networks, GRUD model could achieve convergence fastly. In testing phase, the generator would be separately employed to predict genotype. Kojima model required running 3 RNN models including Lower MAF and Higher MAF model prior to being combined to generate the final results by Hybrid model, while GRUD runs only one RNN model, thereby, the computational time of GRUD reduced significantly.

Moreover, the imputation accuracy of GRUD model could be comparable with Kojima model and HMM-based methods when performing in 1KGP, SG10K, and Vietnamese Genomic datasets derived from different platforms and diverse origins. There was a tendency of lower $R^2$ values in rare variants, especially those imputed by deep-learning-based models which do not take into account the genetic background knowledge, the performance of GRUD model was thereby shown a similar pattern with Kojima model. Moreover, despite the sequential layer improving running time, the imputation accuracy for variants with MAF$\le$0.02 was lower in $R^2$ values compared to the Kojima model in 1KGP data. This is because there are less differences in genotype between samples at these loci, leading to the difficulty for the sequential layer to learn the features and update its weight. Nevertheless, the $R^2$ of GRUD model is considerably higher than others for common variants with MAF>0.05, which accounts for 89,241 of 209,427 variants (42.6%) considered on chromosome 22 in 1KGP phase 3 dataset. In addition to 1KGP data and SG10K, the proposed model was shown comparable or even superior results with other methods for two in-house Vietnamese datasets which were generated by two popular SNP microarrays from Illumina and Affymetrix. Another important advantage of GRUD was the ability to efficiently perform on low-coverage sequencing data. Although the model was evaluated on data from 21 samples of 1KGP, the results showed positive signals, particularly for high-frequency variants, which are paid more attention in downstream analyses^17^. This application could reduce the cost of obtaining genotype data, while still guaranteeing the accuracy of genetic information valuable in clinical practice as well as the implementation of precision medicine. Hence, GRUD might be a potential alternative to traditional methods for genotype imputation on multiple low-cost genotyping techniques.

Currently, the training input of GRUD model needs to have the same shape and fixed positions (e.g., chromosome, start, end) with testing input, which poses several challenges when applying different shapes of input. According to the idea of the masked language model^18,19^, we are looking forward to a trained model with sequencing data that is randomly hidden a number of positions for training in order to allow the model to perform on data generated from different platforms. Besides, we also suggested applying the transfer learning method in the issues of genotype imputation^20^, this could be based on the technique designed with training for a certain task and is used as the foundation for others. It is possible to train the model with genotype data of a chromosome, and then apply it to the remaining others, which could reduce the number of epochs and potentially fit a dataset with small samples in the learning step to improve training time and also performance.

## Methods

### Related work

*Generative adversarial networks (GANs)* GAN architecture comprising two competing neural networks called the Generator—$G$ and the Discriminator—$D$ was firstly introduced in the field of computer vision. From the random noise $z$, $G$ is able to generate a fake data $G(z)$, then the second component—$D$ network makes an effort to differentiate real and fake data from each other. Both networks constantly compete with each other. The generator network attempts to fool the discriminator network. At that point, the discriminator network adapts to the new fake data. This data is then utilized to enhance the generator network, and so forth.

In recent years, a number of authors have proposed applying GAN into language models to address the issues of translation, summarization, etc.^21,22^. In the context of genotype imputation, considering genome as a long string data, the GAN has been potentially used to impute the unobserved variants by utilizing the haplotype reference panel to promote the application of low-cost genotyping approaches, such as SNP microarray chip and low-pass sequencing.

*Recurrent neural network (RNN)* is a popular algorithm for many applications in sequential or time series data thanks to its internal memory in deep learning model architecture^23^. In short, RNN has 2 inputs: the present and the recent past, consequently, the data sequence contains essential information about what will happen next. However, one primary problem of traditional RNN is the difficulty in accessing information computed from a long time ago, which is called vanishing or exploding gradient. Therefore, other types of RNN cells such as Long Short-Term Memory (LSTM)^24^, and Gated Recurrent unit (GRU)^25^ were developed to handle this problem. The gates of these two cell types are capable of learning which data in a sequence should be kept or ignored. Thereby, it could pass relevant information through the long chain of sequences to make predictions. In prior evaluations, due to a simpler structure, GRU was shown to be more computationally efficient than LSTM^26^, so we decided to use GRU cells for our generator model. In specific, the GRU cell consists of 2 main gates: Update gate $\Gamma _u$, and Reset gate $\Gamma _r$ which determine what information should be passed to the next state, and these are also able to create output simultaneously. The general formula of both gates is defined as:

1 $$\begin{aligned} \Gamma =\sigma (Wx_{t} + Ua_{t-1}) \end{aligned}$$

where *W*, *U* are coefficients specific to the gate, the $\sigma$ is the sigmoid function, the $x_t$ is the input of timestep *t*, and the $a_{t-1}$ is the hidden state of timestep before. The information of the new memory will use the reset gate to store information related to the past:

2 $$\begin{aligned} a'_t=tanh(Wx_t+\Gamma _{r} \odot Ua_{t-1}) \end{aligned}$$

where $a'_t$ is a forget value range from $[-1, 1]$. In the final step, the output of the network is to compute $a_t$—the vector containing all the information at time *t* and transmitting it. To do this, the update gate is required and determines what to collect from the current memory—$a'_t$ and information from the previous steps $a_t-1$ :

3 $$\begin{aligned} a_t=\Gamma _u \odot a'_t + (1-\Gamma _u)\odot a_{t-1} \end{aligned}$$

Note that the sign $\odot$ denotes the element-wise multiplication between two vectors.

### Model

The genotype data could be split into two smaller groups: observed variants and unobserved variants. The observed variants are extracted from the genotyping array or sequenced by the low coverage sequencing, while the unobserved variants are not directly identified by genotyping methods, these would be predicted by imputation models. In the imputation process, we assumed both the observed and unobserved variants were sorted according to their positions on the genome. Given the GRU cells we used to construct the model have restricted memory usage, a chromosome was split into different regions, and each region has approximately 1000 observed variants and unobserved variants in our experiments. Noteworthily, observed variants in the upstream and downstream extending section of the region were also included in the input data for the imputation model. Then, the imputation results from all regions are concatenated together in the final result.

**Figure 4:**
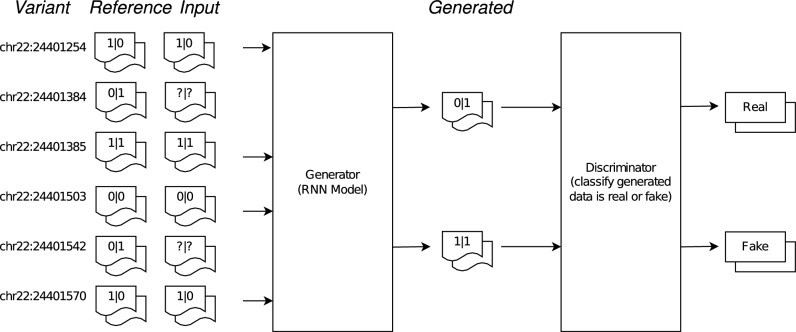
An overview of GRUD model comprising two components: Generator and Discriminator. The generator is a deep network potentially being able to generate unobserved genotypes based on the input of observed variants, and the discriminator tries to distinguish the generated genotype with a haplotype reference panel to determine whether this is a real or fake genotype. RNN: Recurrent neural network.

Assuming that both observed and unobserved variants are biallelic. In other words, their alleles are represented by binary values (0 and 1 for reference (ref) and alternative (alt) allele, respectively). Our model was designed on the basis of GAN architecture comprising two components: Generator and Discriminator (Fig. 4). The generator is a deep network able to generate unobserved genotypes based on the input being observed variants, and the discriminator tries to distinguish the generated genotype with a haplotype reference panel to determine whether this is a real or fake genotype. During the training process, two networks both compete and improve each other at the same time to get better and better imputation accuracy. After that, the generator would be separated from the discriminator to perform imputation with data from SNP microarray chip and low-pass sequencing.

In practical, loss function for training the model over unobserved variants presented in equation 4 is the cumulation of the generator model $L_G$ and the discriminator model $L_D$, which both are binary cross entropy (BCE) between training and reference data.

4 $$\begin{aligned} L = L_D + L_G \end{aligned}$$

#### Generator

**Figure 5:**
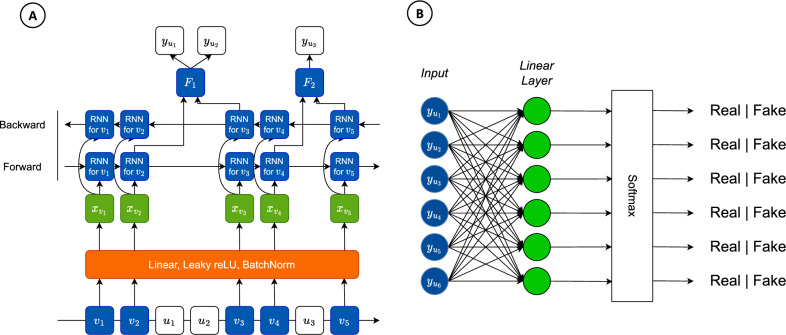
An detailed architecture of GRUD model. (**A**) The structure of the generator model. The proposed model uses a sequential layer before passing bidirectional GRU cells for extracting features. The line in the bottom illustrates a set of variants in their order on the genome where observed variants are in blue rounded square and unobserved are in white ones. Forward and backward RNNs are developed, building off of the observed variants. The input feature of the forward and backward RNNs is $x_{v_i}$ for observed variant $v_i$. With ${\tilde{i}}$ is the index for the closet observed variant *i*, $F_i$ is the vector formed by concatenating the forward RNN’s output for observed variant vi and the output of the backward RNN for the observed variant $v_{i+1}$. $y_{u_i}$ is a binary variable representing the allele for unobserved variant $u_i$. (**B**) Model structure of the discriminator. The input of this model is $y_{u_1},\ldots ,y_{u_n}$ where *n* is the number of unobserved variants, $y_{u_n}$ is the output of the softmax function in the generator.

In the generator (Fig. 5A), from the data of observed variants, we built up a matrix containing allele information of samples in the form of binary data, and this data was transformed into vectors using one-hot encoding, then serving as the input for the further steps. The encoding input was put into a sequential layer which includes a linear layer, activation leakyRelu, and batch norm in order to extract features, add non-linearity, and normalize input into Gaussian distribution, respectively to get the feature matrix $x_v$. After that, a forward GRU is applied on $x_{v_1},\ldots ,x_{v_n}$, and a backward GRU is similar to the forward with $x_{v_n},\ldots ,x_{v_1}$, with *n* is the number of observed variants. With ${\tilde{i}}$ being the index of the unobserved variant for the closest observed variant $v_i$, $F_{{\tilde{i}}}$ are defined as vectors obtained from the concatenation of the output of the forward $o_{forward_i}$ and the output of backward directions $o_{backward_{i+1}}$. The probability of $k=0$ or $k=1$ to know $y_{u_i}$ that is a reference or an alternative is estimated by the softmax function:

5 $$\begin{aligned} softmax(y_{u_{{\tilde{i}}}})=\frac{exp(y_{u_{{\tilde{i}}}})}{\sum _{k=0}^{1}exp(y_{u_{{\tilde{i}}_k}})} \end{aligned}$$

For deep neural networks, vanishing gradients of parameters in backpropagation may lead to failure in parameters training (i.e., the weights of the blocks in the model do not update during the training process). To avoid this problem, we use the residual connection method^27^ that provides a different trackway for data to reach later blocks of the network by skip-connection allowing ensemble data in specific blocks. Therefore, we applied skip-connection to the generator model, which can be obtained by simple changes in the outputs of each layer RNN for $l>1$, with *l*th being the denote of the cardinal number GRU layer; $s_{i,l}$ is the output of GRU cell of observed variant $v_i$ in *l*th GRU layer as follow:

6 $$\begin{aligned} s_{i,l} = s_{i,l}+s_{i, l-1} \end{aligned}$$

Besides, the BCE loss function for training the generator model parameters to calculate the error of the model is denoted:

7 $$\begin{aligned} L_G(z,{\hat{z}})=-\frac{1}{N}\sum _{i=1}^{N}(2MAF_i)^{\gamma }[z_ilog{\hat{z}}_i + (1-z_i)log(1-{\hat{z}}_i)] \end{aligned}$$

where *N* is the number of unobserved variants; ${\hat{z}}_i$ is the predicted probability, while $z_i$ is the reference or alternative label. Besides, the loss function with $\gamma >0$ had a higher priority to higher MAF of unobserved variant *i*, $\gamma <0$ had a higher priority to lower MAF of unobserved variant *i*, and $\gamma =0$ had no priority to MAF, called the Higher, Lower, and Equivalent model, respectively.

#### Discriminator

Reference haplotype panel was considered as real data (positive labels) during training. In addition, we stacked two linear layers which resemble a fully connected layer as hidden layers *f*. With *n* is the number of unobserved variants, the input of the discriminator is $y_{u_1},\ldots ,y_{u_n}$ which is the output of the softmax function in the generator. We summarized the discriminator in Fig. 5B, and the softmax layer was denoted as follow:

8 $$\begin{aligned} softmax(f(y_{u_i}))=\frac{exp(f(y_{u_i}))}{\sum _{k=0}^{1}exp(f(y_{u_{i_k}}))} \end{aligned}$$

We defined BCE loss function for training process as follows:

9 $$\begin{aligned} L_D(p,{\hat{p}})=-\frac{1}{N}\sum _{i=1}^{N}[p_ilog{\hat{p}}_i + (1-p_i)log(1-{\hat{p}}_i)] \end{aligned}$$

where *N* is the number of unobserved samples, the ${\hat{p}}_i$ is the prediction from the discriminator model, while $p_i$ is the real or fake label.

### Experiments

#### Setup and training model

Evaluated model was set up with GRU for RNN cells with 40 hidden units and 8 stacked layers; the feature size of the input vector is 50. The hyperparameters for training process was established with 128 batch size, 100 epochs, 0.001 learning rate, Adam optimizer^28^, early stopping, and exponential learning rate^29^ for decaying the learning rate by every epoch. The metric for evaluating the genotype imputation is R$^2$ value, which is the squared pearson correlation between allele dosages continuously ranged from 0 to 2 (i.e, the value approximately 0,1,2 indicate the genotypes of ref/ref, ref/alt, and alt/alt, respectively) and true genotype counts^30^ as followed:

10 $$\begin{aligned} R^2=\frac{(\sum _{i=1}^{N}(x_i-{\bar{x}})(y_i-{\bar{y}}))^2}{\sum _{i=1}^{N}(x_i-{\bar{x}})^2\sum _{i=1}^{N}(y_i-{\bar{y}})^2} \end{aligned}$$

where $R^2$ is squared pearson correlation, *N* is the number of unobserved variants, $x_i$ and $y_i$ are predicted dosage values and ground truth of unobserved variants *i*, and ${\bar{x}}$ and ${\bar{y}}$ are the means of dosage, respectively. Therefore, “Overall $R^2$” was calculated from whole variants passed into formula 10. In addition, “Average $R^2$ per variant” was implemented on whole datasets following Dias et al. method^12^. We also set up an experiment to investigate the difference of setting with our model by adding the weight to variants based on MAF to the loss function. All of the code was implemented in Python3.8, and PyTorch library version 1.8.1.

#### Datasets

To examine the accuracy of our proposed model and compare its performance to other existing imputation methods on different genotyping platforms including SNP microarray chip and low-pass sequencing, we used the data of chromosome 22 of 1000 genomes project (1KGP) dataset, SG10K, and 1000 Vietnamese genomes project (VN1K) dataset as reference panel. We filtered out variants with MAF<0.005 as the rare variants, which are not usually used for downstream analyses. Details of datasets are summarized in Table 3.

*1000 Genomes Project* The 1KGP phase 3 dataset contains WGS of 2504 people from 5 superpopulations: Africans (AFR), Admixed Americans (AMR), East Asians (EAS), Europeans (EUR), and South Asians (SAS). We simulated genotyping data for designed markers in Infinium Omni2.5-8 Beadchip (Omni2.5) of 100 individuals randomly selected from the 1KGP dataset for evaluating the imputation performance. The remaining 2404 WGS were utilized for constructing the reference panel. The number of variants obtained by Omni2.5 on chromosome 22 is 31,325, and 1,078,043 variants requires imputation in reference panel.

In addition, we downloaded public low-pass sequencing 1KGP data^31^ submitted to the NCBI Gene Expression Omnibus (GEO) under accession number GSE165845 as a test dataset. This dataset included 120 individuals sequenced in three replicates to the target coverage of 1.0x on an Illumina HiSeqX. In the current research, we only investigated 21 samples that intersected with 100 above individuals to save training time for models. The preprocessing data steps were followed by GLIMPSE tutorial^32^ including call variants, and quality control for each sample using BCFtools *mpileup*. After preprocessing, the data contains 283,705 variants as observed data.

*SG10K Consortium* In order to asssess the performance of GRUD for a large dataset, we evaluate our method using SG10K dataset^16^, which consists of genotype profiling of 1,339,154 variants in chromosome 22 obtained by sequencing 4810 Singaporeans (Table 3). We split data into two separate datasets containing 70%, and 30% of the number of samples for training, and testing. Similar to 1KGP phase 3 dataset, the genotyping data of testing dataset was simulated with 26,656 variants of Omni2.5.

*1000 Vietnamese Genomes Project* In addition to 1KGP and SG10K, we also used another in-house dataset, 1000 Vietnamese Genomes Project (VN1K), including the WGS data of 1008 unrelated Vietnamese individuals (https://genome.vinbigdata.org). Participants of the VN1K project were recruited from Kinh ethnic all over Vietnam, from the North to the South, and equally divided among both genders. This dataset obtaining a total of 585,131 variants on chromosome 22, also used as the reference panel.

94 VN1K samples were genotyped by an Axiom™ Precision Medicine Research Array (APMRA), among them, 24 samples were genotyped one more time by the Infinium™ Global Screening Array-24 v3.0 Beadchip (GSAv3) and these genotype data was used to test new imputation method with the true set being their WGS data. The procedure of model training used the data of the remaining 914 samples.

**Table 1:** $R^2$ of the correlation of true genotype and imputed genotype provided by GRUD and other existing models calculated on chromosome 22 variants.

	Omni2.5 -1KGP phase 3		LP-WGS-1KGP phase 3		Omni2.5-SG10K		APMRA		GSAv3	
	Overall $R^2$	Average $R^2$	Overall $R^2$	Average $R^2$	Overall $R^2$	Average $R^2$	Overall $R^2$	Average $R^2$	Overall $R^2$	Average $R^2$
Beagle5.4	0.92088	0.784155	–	–	**0.97395**	**0.23131**	0.94349	0.240624	0.91097	0.16551
Minimac4	0.92922	**0.849513**	–	–	0.96735	0.22661	0.91792	0.238438	0.90853	**0.168622**
GLIMPSE	–	–	0.88667	**0.165236**	–	–	–	–	–	–
Kojima	0.94600	0.831173	–	–	–	–	–	–	–	–
GRUD	**0.94765**	0.819914	**0.90967**	0.0818234	0.96568	0.116065	**0.94462**	**0.416114**	**0.91762**	0.145081

Overall $R^2$: calculated for all variants on chromosome 22 without being separated according to MAF bins.

Average $R^2$: calculated for each variant.

The bold terms indicated the highest values of each column

**Table 2:** Running-time and average memory usage of the GRUD and Kojima model performing in 1KGP with Infinium Omni 2.5-8 Beadchip dataset.

	GRUD	Kojima
Running time (s)	**743**	35,081.99
Average memory usage (GiB)	**0.4389**	2.8215

The bold terms indicated the lowest values of each row

**Table 3:** Summary of reference panels used in comparing genotype imputation performance.

Reference Panel—chr22	Number of samples	Number of variants	Platform	Number of variants in platform
SG10K	3367	1,339,154	Infinium Omni2.5-8 BeadChip	26,656
1KGP phase 3	2404	1,078,043	Infinium Omni2.5-8 BeadChip	31,325
1KGP phase 3	2404	1,078,043	Illumina HiSeqX	283,705
VN1K	914	585,131	Axiom™ Precision Medicine Research Array	11,966
VN1K	914	585,131	Infinium™ Global Screening Array-24 v3.0 BeadChip	8,873

1KGP phase 3: 1000 Genomes Project phase 3; VN1K: 1000 Vietnamese Genomes Project; chr22: chromosome 22

### Ethics declarations

In the VN1K study, subjects provided informed consent and the study was approved by the Vinmec International Hospital Institutional Review Board with number 543/2019/QD-VMEC.

## Data Availability

The 1KGP phase 3 (https://www.internationalgenome.org/), low-pass sequencing data of 1KGP (under accession number GSE165845 in GEO dataset), SG10K (https://www.npm.sg/) and information of SNP array Omni2.5-8 Beadchip (humanomni2.5.8 Beadchip kit) are publicly available on the internet. The VN1K WGS and genotyping datasets are available under agreement at MASH data portal (https://genome.vinbigdata.org/).

## Code availability

The script for calculating averaged R2 per variant is in https://github.com/TorkamaniLab/imputation_accuracy_calculator. Source codes of GRUD model are available from: https://github.com/vinhdc10998/GRUD.
